# CTOS Board of Directors 2000-2001

**DOI:** 10.1155/S1357714X01000019

**Published:** 2001-11


					1357-714X print/1369-1643 online/01/04/S3-01 (c) 2001 Taylor & Francis Ltd
DOI: 10.1080/13577140120048935
Sarcoma (2001) 3, S1, S3-44
CTOS BOARD OF DIRECTORS 2000-2001
Jaap Verweij, MD President
Lee Helman, MD Vice President
Mark Gebhardt, MD Secretary
Robert S. Benjamin, MD Treasurer
Brian O'Sullivan, MD Past President
J. Sybil Biermann, MD (1998-2001)
Vivien Bramwell-Wesley, MD (1998-2001)
Ian Judson, MD (1998-2001)
Claude Turc-Carel, MD (1998-2001)
Frits Van Coevorden, MD (1998-2001)
Franco Gherlinzoni, MD (1999-2002)
Michael Simon, MD (1999-2002)
Scott Nelson, MD (1999-2002)
Martine van Glabbeke, MD (1999-2002)
Ephraim Casper, MD (2000-2003)
Andrew Huvos, MD (2000-2003)
Martin Robinson, MD (2000-2003)
Junya Toguchida, MD (2000-2003)
CTOS abstracts S5
CTOS Meeting Program
Thursday, November 1, 2001
1:00-5:00 p.m. Registration, Set Up Posters
6:00-7:00 p.m. Welcome Reception
Friday, November 2, 2001
7:00 a.m. Registration, Breakfast for meeting attendees
7:00 a.m. Executive Committee Meeting
8:00 a.m. Welcome, Opening Announcements: Jaap Verweij, CTOS President
8:15 a.m. PEDIATRIC SARCOMAS: Chairmen: Lee Helman (USA) & Herbert Jurgens (Germany)
8:15 a.m. Herbert Jurgens: Pediatric sarcoma: state of the art
8:30 a.m. Tim Triche: From gene activation to signal transduction treatment
8:45 a.m. Lenny Wexler: Irinotecan in refractory RMS
9:00 a.m. Peter Anderson: Inhalation GMCSF for treatment of pulmonary
metastases
9:15 a.m. Abstract number 40 Her-2/NEU staining in osteosarcoma: association
with increased risk of metastatis--Holly Zhou, Rob Goldsby, R. Lor
Randall, Lynn Smith, Cheryl M. Coffin Departments of Pathology,
Pediatrics, Orthopedics, and Radiation Oncology, University of Utah
9:30 a.m. Summary and Panel Discussion: Where do we go from here? Lee Helman
10.00 a.m. Coffee Break, Poster Viewing
10:15 a.m. BONE TUMORS: Chairmen: Peter Choong (Australia) & Thor Alvegard (Sweden)
10:15 a.m. Linda Granowetter: US Ewing's sarcoma randomized study of higher dose
therapy
10:30 a.m. Kristy Weber: Implications and management of local relapse in Ewing's
sarcoma
10:45 a.m. Peter Choong: The role of functional nuclear scanning in sarcoma
management
11:00 a.m. Sigbjorn Smeland: An overview of treatments results in osteosarcoma: is
there a need for a global collaboration?
11:15 a.m. Abstract number 87 Reconstruction of the pelvis following resection of
tumors about the acetabulum--R. L. Satcher, Jr., R. J. O'Donnell, J. O.
Johnston, University of California, San Francisco
11:30 a.m. Abstract number 22 Curative treatment and effective palliation with
hemipelvectomy--A useful operation: modern concepts based on 20 years
of experience--James C Wittig, Jacob Bickels, Felasfa Wodajo, Kristen L.
Kellar-Graney, Yehuda Kollender, Isaac Meller, Martin M Malawer
Division of Orthopedic Oncology, Washington Cancer Institute and
National Unit of Orthopedic Oncology, Tel-Aviv Sourasky Medical Center
11:45 a.m. Abstract number 16 (Young Investigator Award Winner) Bugged
out? Infection and endoprosthetic replacements--Lee M Jeys, Raj Suneja,
Vishnu Prasad, Robert Grimer, Simon R Carter, Roger Tillman Royal
Orthopaedic Hospital Oncology Service, Royal Orthopaedic Hospital
12:00 p.m Summary and Panel Discussion: Where do we go from here? Thor Alvegard
12:15 p.m. Buffet Lunch, Poster Viewing
12:15 p.m Board of Directors Meeting
1:15 p.m. Jaap Verweij, CTOS President: Presentations of Two Young Investigator Awards
(Lee M Jeys, Jonathan C Trent)
S6 CTOS abstracts
1:30 p.m. SOFT TISSUE SARCOMAS: Chairmen: Jean-Yves Blay (France) & Brian O'Sullivan
(Canada)
1:30 p.m. Pancras Hogendoorn: Grading of soft tissue tumours
1:45 p.m. Peter Pisters: Preoperative chemoradiotherapy for locally advanced soft
tissue sarcomas
2:00 p.m. Brian O'Sullivan: The advantage of new radiotherapy technologies in the
management of soft tisssue sarcoma
2:15 p.m. Controversy session: polychemotherapy vs. monochemotherapy in
advanced sarcomas:
2:15 p.m. pro: Bob Benjamin
2:30 p.m. contra: Jaap Verweij
2:45 p.m. Discussion
3:00 p.m. Abstract number 56 Margin width has prognostic significance for
extremity and truncal sarcoma--Mark D.McKee, Dong Feng Liu,
Deborah Driscoll, John J. Brooks, John F. Gibbs, William G. Kraybill
Roswell Park Cancer Institute
3:15 p.m. Summary & Panel Discussion: Where do we go from here? Jean Yves Blay
3:45 p.m. Coffee Break, Poster Viewing
4:00 p.m. BASIC SCIENCE/BIOLOGY: Jonathan Fletcher (USA) & Junya Toguchida (Japan)
4:00 p.m. Marc Ladanyi: Alveolar soft part sarcoma oncogene
4:20 p.m. Charlie Roberts: SNF5/INI1 haploinsufficiency predisposes to rhabdoid
tumors in mice
4:40 p.m. Paul Meltzer: Clinical and biological applications of sarcoma gene
expression profiling
5:00 p.m. Abstract number 106 Gene-expression profiles in chondrosarcomas and
chordomas--Thomas F. DeLaney, Brian Seed, Ramnik Xavier, Andrew
L. Rosenberg, G. Petur Nielsen, Francis J. Hornicek, Norbert J. Liebsch,
John E. Munzenrider, Herman D. Suit, Massachusetts General Hospital
5:15 p.m. Summary and Panel discussion: Jonathan Fletcher & Junya Toguchida
6:00-6.30 p.m. Members' Business Meeting
7:15 p.m CTOS Reception and Dinner Banquet
Saturday, November 3, 2001
7:00 a.m. Registration, Breakfast for meeting attendees
8:00 a.m. NINA AXELRAD KEYNOTE LECTURE (Supported by the Nina Axelrod Sarcoma
Fund):
Allan van Oosterom (Leuven, Belgium): Systemic treatment of advanced soft tissue sarco-
mas is not uniform any longer; Glivec as a paradigm.
9:00 a.m. IMATINIB MESYLATE (DEVELOPED AS STI-571): George Demitri (USA) & Jaap
Verweij (Netherlands)
9:00 p.m. Brian Rubin: Biology of GISTs
9:15 p.m. George Demitri: The USA experience
9:30 p.m. Jaap Verweij: The EORTC phase 2 study
9:45 p.m. Abstract number 131 (Young Investigator Award Winner)
Inhibition of PDGF receptor tyrosine kinase in human soft-tissue
sarcoma--Jonathan C. Trent, Jonathan Hannay, Lan Li, Shreyaskumar
Patel, Robert S. Benjamin, Raphael E. Pollock, Di-Hua Yu The
University of Texas, M. D. Anderson Cancer Center
10:00 p.m. Panel Discussion: Where do we go from here? George Demitri & Jaap
Verweij
CTOS abstracts S7
10:15 a.m. Coffee Break, Poster Viewing
10:30 a.m. Workshop: Genetics of Sarcoma: Jonathan Fletcher (USA) & Lee Helman (USA)
10:30 a.m. Paola Dal Cin: Novel cytogenetic mechanisms in mesenchymal tumors
10:50 a.m. Marc Ladanyi: Specificity and clinical correlations for SYT-SSX
11:10 a.m. Abstract number 130 Expression profiling of osteosarcoma biopsies and
metastases with CDNA microarrays--Luca Sangiorgi, Giuliana
Alessandra Gobbi, Enrico Lucarelli, Francesca Scrimieri, Piero Picci, Paul
Meltzer, Lab. Oncology Research, Rizzoli Orthopedic Institute, and
Section of Molecular Genetics, Cancer Genetics Branch, National
Human Genome Research Institute, National Institutes of Health
11:25 a.m. Summary and Panel Discussion: Where do we go from here? Jonathan
Fletcher & Lee Helman
11:45 a.m. Buffet Lunch, Poster Viewing
12:45 p.m. COOPERATIVE GROUP RESEARCH: Jaap Verweij (The Netherlands) & Karen
Antman (USA)
1:45 p.m. Mini Symposium: STAGING, RESPONSE ASSESSMENT & FOLLOW-UP: Peter
Hohenberger (Germany) & Ian Judson (UK)
1:45 p.m. Jan Bloem: Staging and dynamic contrast enhanced MRI for response
assessment
2:00 p.m. Peter Hohenberger: 31P magnetic resonance spectroscopy (31P-MRS)
for non-invasive assessment of pathohistologic tumor response of soft
tissue sarcoma
2:15 p.m. A. Kevin Raymond: Pathohistological response assessment following
systemic chemotherapy for sarcomas
2:30 p.m. Summary and Panel Discussion: Where do we go from here? Ian Judson
2:45 p.m. CASE DISCUSSIONS: ANGIOSARCOMAS Paolo Casali (Italy)
Medical Oncology: Paolo Casali
Pathology: John Brooks
Surgery: Sylvie Bonvalot
Radiotherapy: Kirsten Sundby Hall
3:45 p.m. Adjournment

				

